# Nuclear Import of Transcription Factor BR-C Is Mediated by Its Interaction with RACK1

**DOI:** 10.1371/journal.pone.0109111

**Published:** 2014-10-03

**Authors:** Daojun Cheng, Wenliang Qian, Yonghu Wang, Meng Meng, Ling Wei, Zhiqing Li, Lixia Kang, Jian Peng, Qingyou Xia

**Affiliations:** 1 State Key Laboratory of Silkworm Genome Biology, Southwest University, Chongqing, China; 2 School of Life Science, Southwest University, Chongqing, China; 3 Laboratory of Silkworm Science, Kyushu University Graduate School of Bioresource and Bioenvironmental Sciences, Fukuoka, Japan; Wuhan University, China

## Abstract

The transcription factor Broad Complex (BR-C) is an early ecdysone response gene in insects and contains two types of domains: two zinc finger domains for the activation of gene transcription and a Bric-a-brac/Tramtrack/Broad complex (BTB) domain for protein-protein interaction. Although the mechanism of zinc finger-mediated gene transcription is well studied, the partners interacting with the BTB domain of BR-C has not been elucidated until now. Here, we performed a yeast two-hybrid screen using the BTB domain of silkworm BR-C as bait and identified the receptor for activated C-kinase 1 (RACK1), a scaffolding/anchoring protein, as the novel partner capable of interacting with BR-C. The interaction between BR-C and RACK1 was further confirmed by far-western blotting and pull-down assays. Importantly, the disruption of this interaction, via RNAi against the endogenous *RACK1* gene or deletion of the BTB domain, abolished the nuclear import of BR-C in BmN4 cells. In addition, RNAi against the endogenous *PKC* gene as well as phosphorylation-deficient mutation of the predicted PKC phosphorylation sites at either Ser373 or Thr406 in BR-C phenocopied *RACK1* RNAi and altered the nuclear localization of BR-C. However, when BTB domain was deleted, phosphorylation mimics of either Ser373 or Thr406 had no effect on the nuclear import of BR-C. Moreover, mutating the PKC phosphorylation sites at Ser373 and Thr406 or deleting the BTB domain significantly decreased the transcriptional activation of a BR-C target gene. Given that RACK1 is necessary for recruiting PKC to close and phosphorylate target proteins, we suggest that the PKC-mediated phosphorylation and nuclear import of BR-C is determined by its interaction with RACK1. This novel finding will be helpful for further deciphering the mechanism underlying the role of BR-C proteins during insect development.

## Introduction

The Broad-Complex (BR-C) protein contains a pair of C2H2 zinc fingers at the C terminus and a Bric-a-brac/Tramtrack/Broad complex (BTB) domain at the N terminus [1], [2]. In insects, BR-C is primarily involved in the signaling pathways of ecdysone and juvenile hormone, two key endocrine hormones that orchestrate the growth and development of insects [3]. Accumulated evidence has demonstrated the critical roles of insect BR-C in numerous developmental processes, including embryogenesis [4], [5], metamorphosis [6], [7], cell death [8], [9], stem cell differentiation [10], wing morphogenesis [11], eye development [12], muscle attachment [13], [14], metamorphic remodeling of the central nervous system [15], [16], and oogenesis [17], [18].

It has been reported that the transcription of insect *BR-C* gene is activated and suppressed by ecdysone and juvenile hormone, respectively [19]. In particular, the BR-C protein is considered an early response genes in the ecdysone signaling pathway, regulating the transcription of downstream target genes, such as cuticle protein genes [20]–[22], microRNA genes [23], [24], caspase genes [25], glue protein genes [26], and fork head genes [27].

Although zinc fingers-mediated transcriptional regulation for BR-C protein is well understood in insects, the role of BTB domain of BR-C protein remains unknown. Given that the BTB domain is a conserved motif involved in protein-protein interaction [28], it is expected that the BTB domain would provide the BR-C protein with a facile means of interacting with some partners. In fact, several BTB domain-containing proteins have been identified to interact with partners via their BTB domain [29]–[34],

Silkworm (*Bombyx mori*) is regarded as an excellent model system for the lepidopterans [35]. The expression profile, target gene, and function during metamorphosis of the silkworm *BR-C* gene have all been characterized [8], [20], [36]. To identify potential partners interacting with BR-C protein, we performed a yeast two-hybrid screen using the BTB domain of the silkworm BR-C protein as bait. Ultimately, we identified a novel interacting partner for silkworm BR-C, receptor for activated C-kinase 1 (RACK1), which has been previously demonstrated to function an anchoring protein for activated protein kinase C (PKC) [37], [38] and a scaffolding protein for recruiting PKC to a number of binding partners to trigger their PKC-mediated phosphorylation [39]–[42]. Further examination revealed that the interaction between BR-C and RACK1 is required for the nuclear import of BR-C.

## Materials and Methods

### Yeast two-hybrid screen

The Matchmaker Gold Yeast Two-Hybrid System (Clontech) was used to perform the yeast two-hybrid screen in this study. All experiments were carried out according to the standard protocol for this system. Briefly, the cDNA sequence encoding the BTB domain of the silkworm BR-C protein (GenBank no: NP_001104803) was amplified through a standard PCR approach and then cloned into a pGBKT7 vector to generate the bait plasmid pGBKT7-BTB. The primers and plasmid constructs are listed in Table S1 and Table S2. The pGBKT7-BTB construct was transformed into Y2HGold yeast to obtain the bait strain. Given that the BR-C gene is abundantly expressed in the silkworm silk gland [36], we extracted total RNA from the silk gland using the TRIzol reagent (Invitrogen) to construct a cDNA library for preying the BTB-interacting proteins. Strains containing the pGBKT7-BTB bait plasmid and the cDNA library were co-cultured in YPDA liquid media for yeast mating and then grown on dropout medium (SD/-Leu/-Trp) lacking both Leu and Trp to initially select positively interacting clones. We isolated and cultured these clones on selective dropout medium (SD/-Ade/-His/-Leu/-Trp/X-a-Gal), which lacks four amino acids (Ade, His, Leu, and Trp) and contains X-a-Gal as a reporter, to more stringently select positively interacting clones. These positive clones were individually collected to isolate their plasmid DNA using the Easy Yeast Plasmid Isolation Kit (Clontech), and each plasmid was sequenced and annotated based on NCBI BLAST searches.

### Cell culture

Two silkworm ovary-derived cell lines, BmN4 cells and BmN4-SID1 cells harboring the *Caenorhabditis elegans SID1* gene [24], were used in this study. The cells were cultured at 27°C in IPL-41 medium (Sigma, USA) supplemented with 10% fetal bovine serum (Life Technologies, USA).

### Subcellular localization

For subcellular localization assays, full-length cDNAs encoding the silkworm BR-C and RACK1 (GenBank no: NP_001041703) genes were separately cloned into the expression vector pi2VW (a vector containing the green fluorescent protein variant Venus) to generate the Venus-BR-C and Venus-RACK1 plasmids according to the previously described procedure [43]. Meanwhile, we also generated a construct (BR-C-DsRed) containing the full-length silkworm BR-C gene fused to DsRed (a red fluorescent protein reporter) gene to examine the nuclear localization of the silkworm BR-C protein.

The plasmid constructs Venus-RACK1, Venus-BR-C and BR-C-DsRed were transfected into BmN4 cells in 12-well plates at 100 ng per well. At 48 h after transfection, the BmN4 cells were seeded onto coverslips. After washing with PBS, the cells were fixed with 4% paraformaldehyde for 15 min and permeabilized with 0.1% TritonX-100 for 15 min. BmN4 cells were then stained with DAPI (1∶1,000) for 15min, washed three times with PBS, and the coverslips were mounted on glass slides. Fluorescence signals were checked and captured via confocal microscopy (Fv1000, Olympus).

### Expression and purification of recombinant proteins

The full-length silkworm *BR-C* gene was cloned into the vectors pET28-His and pCold-SUMO containing a His tag. The full-length silkworm *RACK1* gene was cloned into the pETGST vector. After sequencing, each recombinant vector was transformed into *E. coli* strain BL21 and induced for 20 h by 0.2 mM IPTG at 16°C. The cells were harvested and lysed by sonication. GST and GST fusion proteins were purified using glutathione-conjugated agarose beads. The purified His-BR-C protein was used to produce a rabbit antibody against BR-C (Zoonbio Biotechnology).

### Far-western blotting assay

The interaction between BR-C and RACK1 was first confirmed in vitro by far-western blotting experiments [44]. BSA, GST, and GST-RACK1 protein samples (5 µg per sample) were separately electro-transferred onto a 0.45 µm PVDF membrane. After denaturation and renaturation, the membrane was blocked with buffer containing 5% skim milk powder (Boster) and incubated with SUMO-BR-C proteins (50 µg) overnight at 4°C. Another control was prepared by the incubating the SUMO protein with GST-RACK1. Antibodies against GST (Sigma; 1∶100) and silkworm BR-C (1∶1,000) were used for western blotting.

### GST pull-down assay

Glutathione agarose beads (25 µl) coated with either GST or the GST-RACK1 fusion protein (5 µg per sample) were mixed with SUMO or SUMO-BR-C fusion proteins (50 µg per sample) in 1 ml PBS buffer (pH 8.0). Each mixture was incubated with shaking for 8 h at 4°C. The beads were collected by centrifugation and washed three times in PBS buffer and then eluted with 80 µl 50 mM Tris-HCl (pH 8.0) containing 10 mM GSH to isolate the supernatant. The supernatants were mixed with sample loading buffer and separated on SDS-PAGE gels and immunoblotted with anti-His (Sigma; 1∶100) or anti-BR-C antibodies. In addition, we also used the GST-BR-C fusion protein to pull down RACK1 that was prepared from BmN4 cells. The proteins in the supernatants were immunoblotted with anti-RACK1 antibody (Cell signaling technology; 1∶100).

### RNA interference

RNA interference (RNAi) was used to knockdown the expression of target genes. Double-strand RNA (dsRNA) targeting silkworm *RACK1*, *PKC* (GenBank no: NM_001043513), or *EGFP* (enhanced green fluorescent protein) as a control was synthesized according to the RiboMAX Large Scale RNA Production System-T7 (Promega). BmN4-SID1 cells were treated with different dsRNAs as previously described [43]. Briefly, the dsRNA for each gene was added to the medium to induce RNAi. After two rounds of dsRNA treatment, the BR-C-DsRed plasmid was transfected. At 48 h after transfection, the subcellular localization of the BR-C protein was examined via a confocal microscopy.

### Site-directed mutagenesis and BTB domain deletion

The full-length silkworm *BR-C* gene inserted into the pMD19-T vector was used as a template for mutating the PKC phosphorylation sites or deleting the BTB domain using the MutanBEST Kit (Takara). For phosphorylation-deficient mutation, each of the predicted phosphorylation sites, namely, Ser (S) or Thr (T), was mutated to Ala (A). For phosphorylation mimics, the predicted phosphorylation sites were singly mutated to Glu (E). The modified *BR-C* gene sequences were subcloned into a plasmid harboring the DsRed reporter to generate constructs containing the BR-C mutations or the BTB deletion fused to DsRed. Each construct was also used to check the subcellular localization as described above.

### Luciferase reporter assay

Given that the silkworm wing cuticle protein gene *WCP10* is a direct target of BR-C [20], the effects of PKC phosphorylation sites mutations or BTB deletion in BR-C on the transcription of the *WCP10* gene were analyzed using the dual luciferase reporter assay system (Promega). The regulator region from −2940 to +26 in the *WCP10* promoter was cloned into a pGL3 basic vector containing a dual luciferase reporter. The DsRed-BR-C, DsRed-BR-C mutants, and DsRed-BTB deletion constructs were separately co-transfected into BmN4 cells with the WCP10-lux reporter vector (1 ug per well). The cells were cultured at 27°C for 48 h. Luciferase activities were measured according to the manufacturer's protocol for the luciferase reporter system.

## Results

### Identification of RACK1 as a BR-C-interacting protein by yeast two-hybrid screen

**Figure 1:**
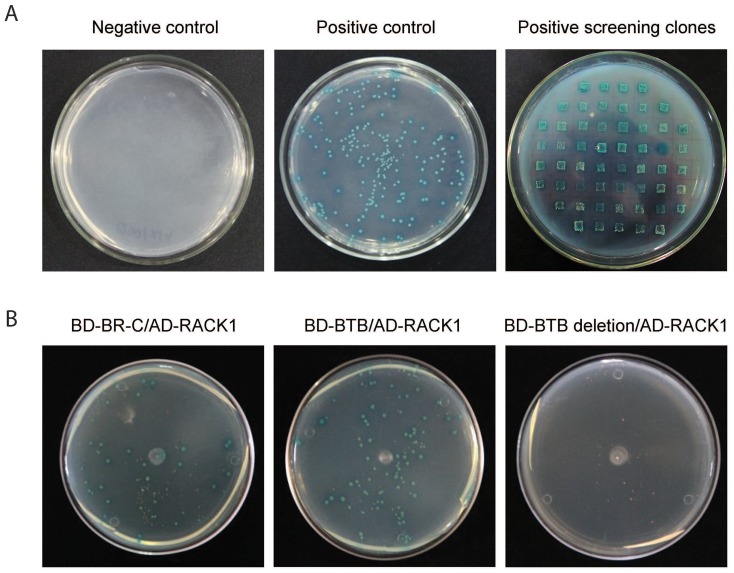
Identification of BR-C-interacting proteins through a yeast two-hybrid screen. (A) Yeast two-hybrid screen of BR-C-interacting proteins from a silkworm silk gland cDNA library using the BTB domain of BR-C as bait. A total of 54 positive clones were found to grow on selective medium. A negative control consisting of pGBKT7-Lam with pGADT7-T and a positive control consisting of pGBKT7-53 with pGADT7-T were used to measure the efficiency of the system. (B) Yeast two-hybrid confirmation of the interaction between BR-C and its novel interacting partner RACK1. cDNA encoding full-length BR-C, the BTB domain or partial BR-C lacking the BTB domain was cloned into the pGBKT7 vector. The full-length cDNA sequence of *RACK1* gene was inserted into the pGADT7 vector. The yeast two-hybrid experiment was performed to test the interaction between BR-C and RACK1.

Insect BR-C transcription factors contain a conserved BTB domain, which is a protein-protein interacting motif [2]. To identify partners interact with the BR-C protein, we performed a yeast two-hybrid screen of a silkworm silk gland cDNA expression library using the BTB domain of the silkworm BR-C protein as bait. A preliminary screen identified 54 positive clones capable of growing on selective dropout medium (Figure 1A). Sequencing result revealed that 17 of these positive clones encode RACK1 (GenBank no: NP_001041703), indicating that RACK1 may be an interacting partner for the silkworm BR-C protein.

To confirm the interaction between BR-C and RACK1 in yeast cells, we obtained three silkworm *BR-C* gene sequences, including the full-length cDNA, the BTB domain sequence alone, and a partial cDNA sequence lacking the BTB domain, which were then fused to the GAL4 DNA binding domain (GAL4BD) to produce the bait constructs BD-BR-C, BD-BTB, and BD-BTB deletion. In addition, the complete coding sequence for the silkworm RACK1 gene was fused to GAL4 activation domain (GAL4AD) to generate the prey construct AD-RACK1. Two-hybrid analysis showed that RACK1 could efficiently interact with both full-length BR-C and the BTB domain alone, but not a partial BR-C protein lacking the BTB domain (Figure 1B), indicating the BTB domain of BR-C was necessary for the interaction between BR-C and RACK1.

### BR-C interacts with RACK1 in vitro

**Figure 2:**
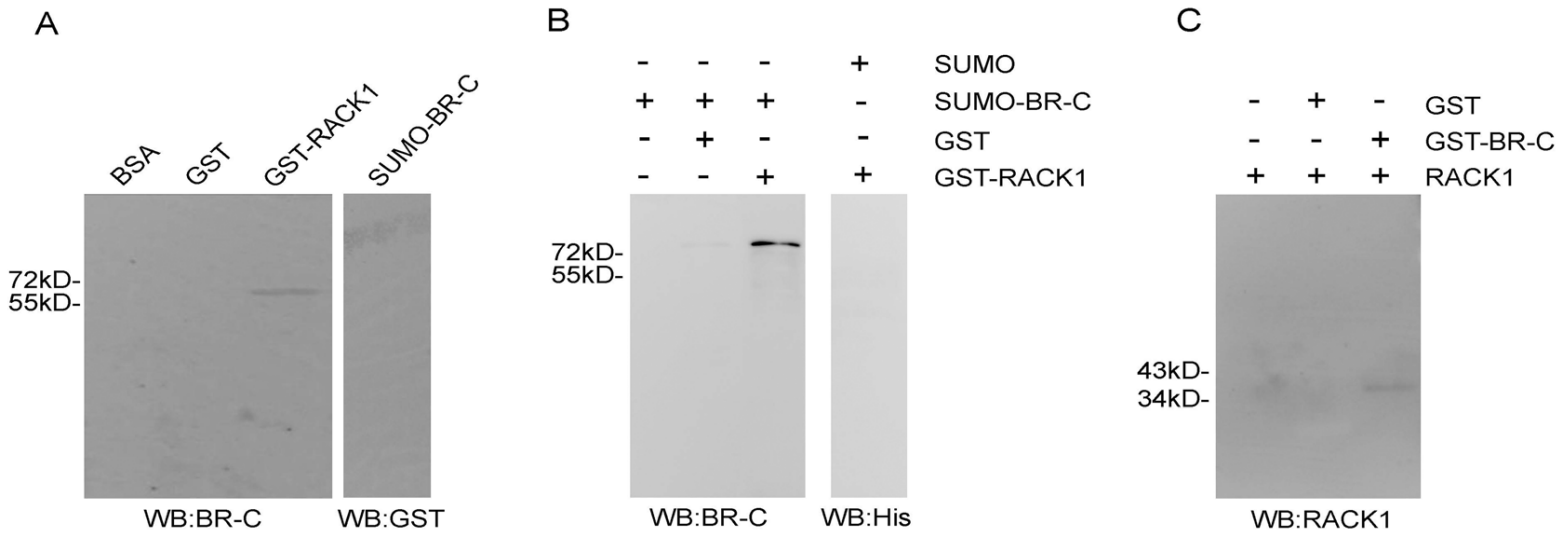
Interaction of BR-C with RACK1 in vitro. (A) Interaction between BR-C and RACK1 by far-western blotting. GST, GST-tagged RACK1 (GST-RACK1), and SUMO-tagged BR-C (SUMO-BR-C) were expressed and purified from prokaryotic cells. The purified proteins were separated on 12% SDS-PAGE gels and transferred onto PVDF membranes, which were incubated with SUMO-BR-C or GST proteins at 4°C overnight, and analyzed by western blotting using anti-BR-C or anti-GST antibodies. (B-C) Interaction between BR-C and RACK1 by GST pull-down assay. Purified SUMO-BR-C was incubated with purified GST-RACK1 and examined by immunoblotting using an anti-BR-C antibody (B). Precleared BmN4 cell lysates containing endogenous RACK1 were incubated with purified GST-BR-C at 4°C for 6 h, and were separated via 12% SDS-PAGE gel and visualized by immunoblotting using an anti-RACK1 antibody (C).

To further confirm the interaction of BR-C with RACK1 in vitro, we first conducted a far-western blotting analysis using bacterially expressed GST-tagged RACK1 (GST-RACK1) and SUMO-tagged BR-C (SUMO-BR-C). As shown in Figure 2A, BR-C was able to interact with RACK1 but not with GST only.

The interaction between BR-C and RACK 1 was also confirmed by a GST pull-down assay. The SUMO-tagged BR-C protein could be pulled down using GST-tagged RACK1 but not by GST alone, and bacterially expressed SUMO could not be pulled down by GST-tagged RACK1 (Figure 2B). Similarly, the endogenous RACK1 protein extracted from BmN4 cells could be pulled down by GST-tagged BR-C but not GST alone (Figure 2C). These data demonstrate a direct interaction between BR-C and RACK1.

### BR-C and RACK1 localize to different subcellular regions in BmN4 cells

**Figure 3:**
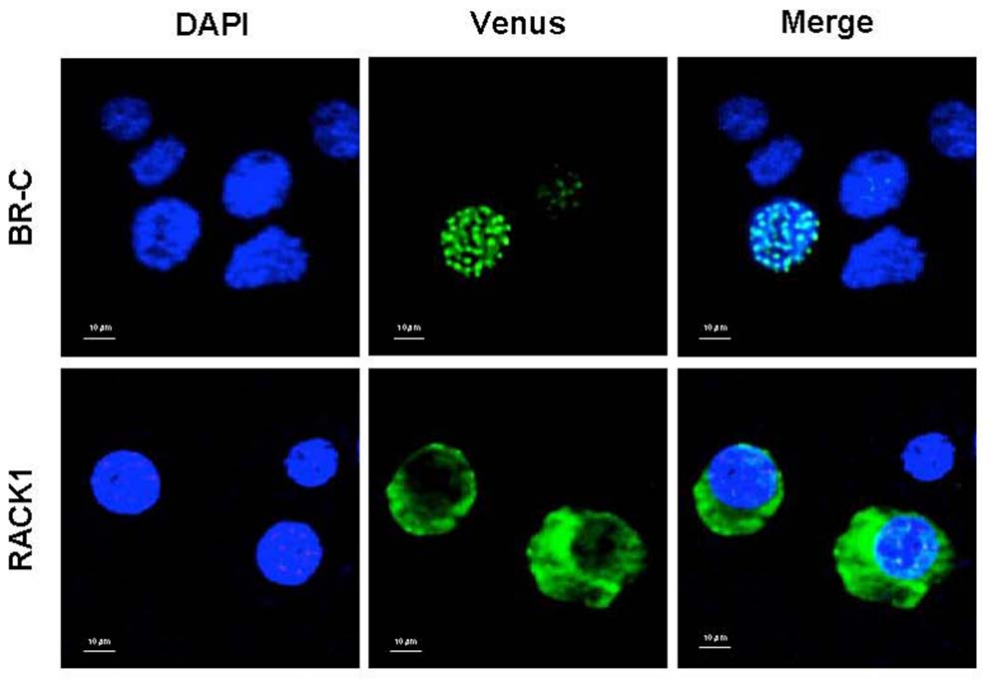
Subcellular localization of BR-C and RACK1. RACK1 fused with Venus (Venus-RACK1) and BR-C fused with Venus (Venus-BR-C) were separately transfected into BmN4 cells. Two days post transfection, the cells were fixed and observed using confocal microscopy. The DNA was stained with DAPI.

To understand how BR-C interacts with RACK1 in vivo, we surveyed the subcellular localization of BR-C and RACK1 in BmN4 cells. Venus-fused BR-C and RACK1 were individually transfected into BmN4 cells. After two days of transfection, the cells were observed via confocal fluorescence microscopy. Venus-BR-C was found to localize exclusively in the nucleus in BmN4 cells, forming multiple spots (Figure 3), which was expected due to the role of BR-C as a transcription factor. However, Venus-RACK1 was mainly localized diffusely throughout the cytoplasm of BmN4 cells (Figure 3). Combined with the fact that RACK1 generally acts as a scaffolding/anchoring protein to coordinate the PKC-mediated phosphorylation of binding partners [45], [46], we therefore hypothesized that the interaction between BR-C and RACK1 may occur transiently in the cytoplasm and further contribute to the nuclear localization of BR-C or even to its posttranslational modification.

### BR-C BTB domain deletion and *RACK1* RNAi both disrupt BR-C nuclear localization

**Figure 4:**
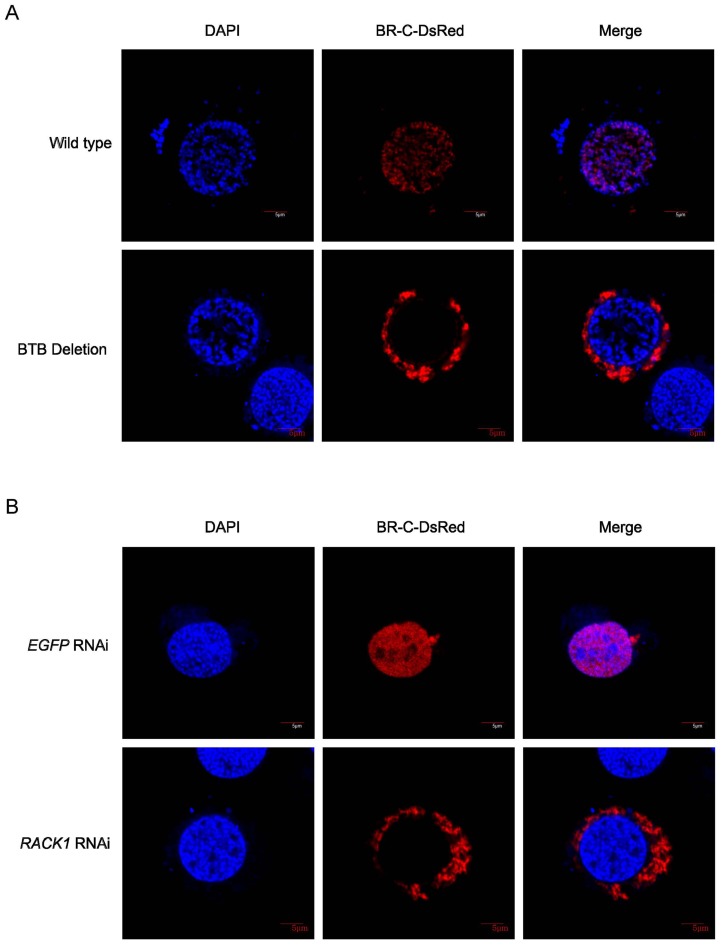
Disrupting the interaction between BR-C and RACK1 affects the nuclear import of BR-C. (A) BTB domain deletion affects the nuclear localization of exogenously expressed BR-C in BmN4 cells. Constructs expressing full-length BR-C and partial BR-C lacking the BTB domain were fused to DsRed and transfected into BmN4 cells, and the cells were observed by confocal microscopy two days after transfection. (B) RNAi against endogenous *RACK1* gene inhibits the nuclear import of exogenously expressed BR-C in BmN4-SID1 cells. Double-stranded RNA (dsRNA) targeted the *RAC*K1 gene or the control *EGFP* gene was added to the culture medium separately for BmN4-SID1 cells, and five days later, the plasmid expressing full-length BR-C fused to DsRed was transfected into BmN4-SID1 cells. Confocal microscopy analysis was performed two days after transfection. Treatment with dsRNA against enhanced green fluorescent protein (*EGFP*) gene was used as a control. DAPI was used to stain the nuclear DNA of the cells.

To test the hypothesis that the interaction between BR-C and RACK1 may affect the nuclear localization of BR-C, we disrupted the RACK1-BR-C interaction by either deleting the BTB domain of BR-C or performing RNAi against *RACK1*. For this analysis, we used the fusion protein construct BR-C-DsRed, in which full-length BR-C was inserted in the upstream of the red fluorescent protein DsRed. Consistent with the localization of Venus-BR-C, DsRed-BR-C was also localized to the nucleus (Figure 4A). Interestingly, DsRed fused to a truncated BR-C protein lacking the BTB domain was retained in the cytoplasm of BmN4 cells (Figure 4A).

Moreover, we performed RNAi-mediated expression silencing of the endogenous *RACK1* gene in BmN4-SID1 cells. In contrast to control cells treated with the treatment with dsRNAs against the *EGFP* gene as a control, full-length BR-C fused to DsRed could not enter the nucleus and was restricted to the cytoplasm in BmN4 cells treated with dsRNAs against the *RACK1* gene (Figure 4B), similar to the effects caused by deletion of the BR-C BTB domain. These results strongly demonstrated that the nuclear localization of BR-C is regulated by its interaction with RACK1.

### Both *PKC* RNAi and PKC phosphorylation site mutations in BR-C alter BR-C nuclear localization

**Figure 5:**
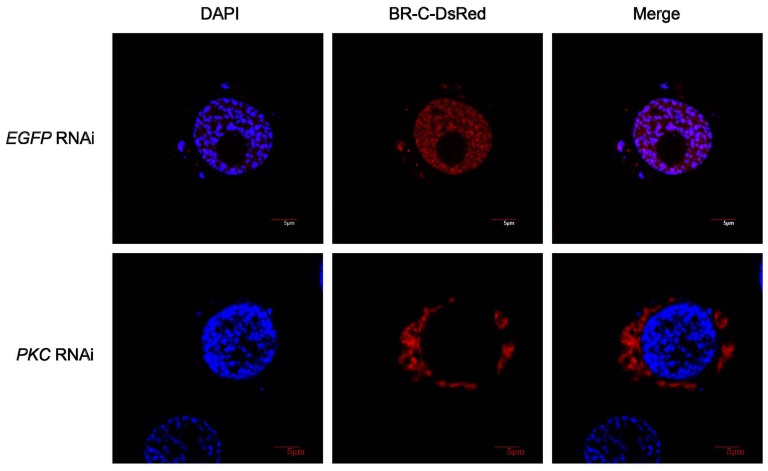
RNAi against the endogenous *PKC* gene alters the nuclear import of BR-C. dsRNA against the *PKC* gene was used to treat BmN4-SID1 cells. Five days later, the construct expressing full-length BR-C fused to DsRed was transfected into BmN4-SID1 cells. The nuclear import of exogenously expressed BR-C was checked at two days after transfection using confocal microscopy. *EGFP* RNAi was used as a control.

We next asked whether the nuclear localization of BR-C is associated with PKC-mediated phosphorylation that is regulated by RACK1. To test this hypothesis, we first performed RNAi against endogenous *PKC* in BmN4-SID1 cells. As shown in Figure 5, *PKC* RNAi resulted in a change in the cellular localization of BR-C from the nucleus to the cytoplasm, which phenocopied the effect of *RACK1* RNAi.

**Figure 6:**
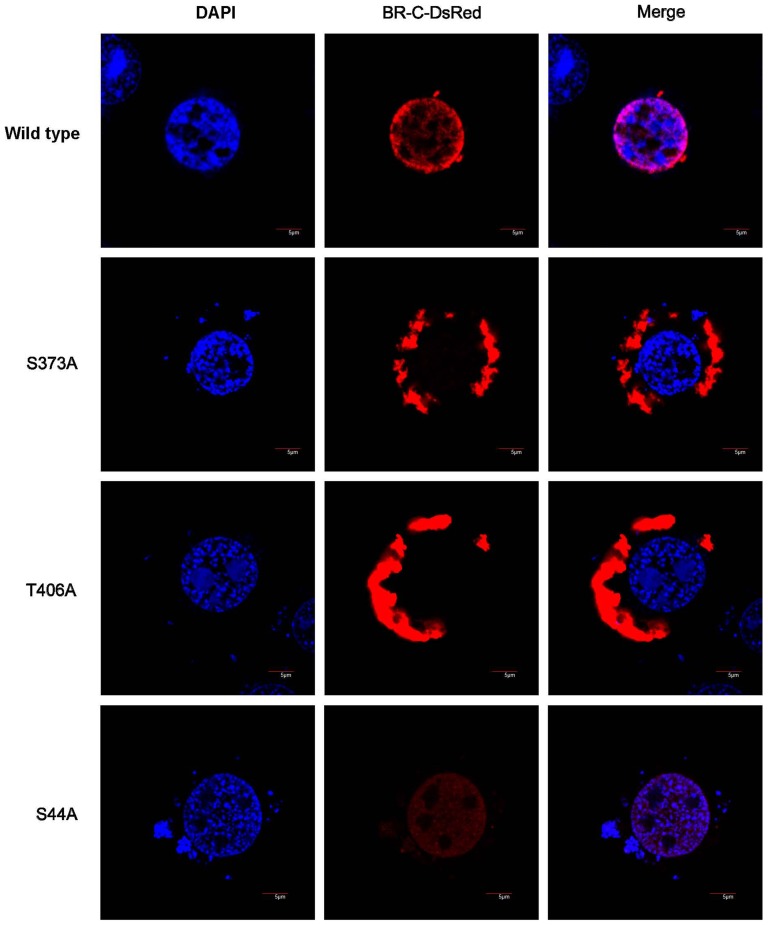
Mutating the predicted PKC phosphorylation sites in BR-C disrupts the nuclear import of BR-C. Each construct expressing full-length BR-C with the mutation of a single predicted phosphorylation site to Ala (A; blocks phosphorylation) was transfected into BmN4 cells, and the nuclear import of the expressed BR-C protein was observed by confocal microscopy two days after transfection. The construct encoding wild-type BR-C without any phosphorylation site mutation was used as a control. Two predicted phosphorylation site mutations, S373A and T406A, led to the failure of BR-C nuclear import. However, mutating the predicted PKC phosphorylation site at Ser44 could not affect the nuclear localization of BR-C.

Next, we analyzed whether mutating PKC phosphorylation sites in the BR-C protein could also affect the nuclear localization of BR-C. Based on the online NetPhosK program (http://www.cbs.dtu.dk/services/NetPhosK/), we predicted 12 PKC phosphorylation sites in the silkworm BR-C protein, including seven Ser (S) and five Thr (T) residues, of which seven sites are located in either BTB domain or zinc finger domains (Figure S1). We generated a series of BR-C mutants in which a single phosphorylation site (Ser or Thr) was specifically mutated to Ala (A), which can block phosphorylation, and then fused these to DsRed. The localization of these mutant fusion proteins was analyzed in BmN4 cells via confocal microscopy. We found that mutations at two sites in the zinc finger motifs, Ser373 and Thr406, clearly shifted the nuclear localization of BR-C to the cytoplasm (Figure 6). By contrast, mutations at Ser44 and at other predicted PKC phosphorylation sites could not affect the nuclear localization of BR-C (Figure 6 and Figure S2). Therefore, these data suggested that PKC is involved in the nuclear import of BR-C, likely through the phosphorylation of BR-C at Ser373 and Thr406.

### Phosphorylation mimics of the PKC phosphorylation sites in BR-C lacking BTB domain have no influence on BR-C nuclear localization

**Figure 7:**
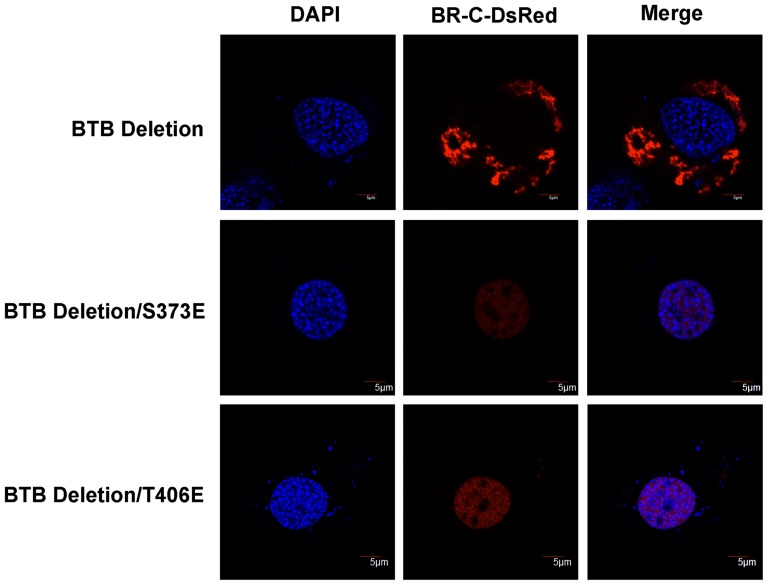
Mutations of phosphorylation sites to Glu in BR-C lacking BTB domain have no impact on nuclear import of BR-C. Construct expressing BR-C both lacking BTB domain and mutating the predicted phosphorylation sites of either Ser373 or Thr406 into Glu (E; mimics phosphorylation) was transfected into BmN4 cells. Confocal microscopy analysis revealed that compared to the control of BR-C only lacking BTB domain, the mutants of either S373E or T406E in BR-C without BTB domain could not change nuclear localization of BR-C.

To further verify whether the interaction of BTB domain of BR-C with RACK1 is necessary for phosphorylation-mediated nuclear import of BR-C, the predicted PKC phosphorylation sites of either Ser373 or Thr406 in BR-C laking BTB domain were mutated to Glu (E), which mimics phosphorylation, and their effects on nuclear import of BR-C were examined in BmN4 cells. As shown in Figure 7, although BTB domain was deleted, either S373E or T406E mutants did not disrupt the nuclear import of BR-C lacking BTB domain. This result, together with the observation that the phosphorylation-deficient mutations at either Ser373 or Thr406 abolish the nuclear import of BR-C, suggested that the interaction of BTB domain in BR-C with RACK1 is primarily required for PKC-mediated phosphorylation and subsequent nuclear import of BR-C.

### BTB deletion and phosphorylation site mutations in BR-C suppress the transcriptional activation of target gene

**Figure 8:**
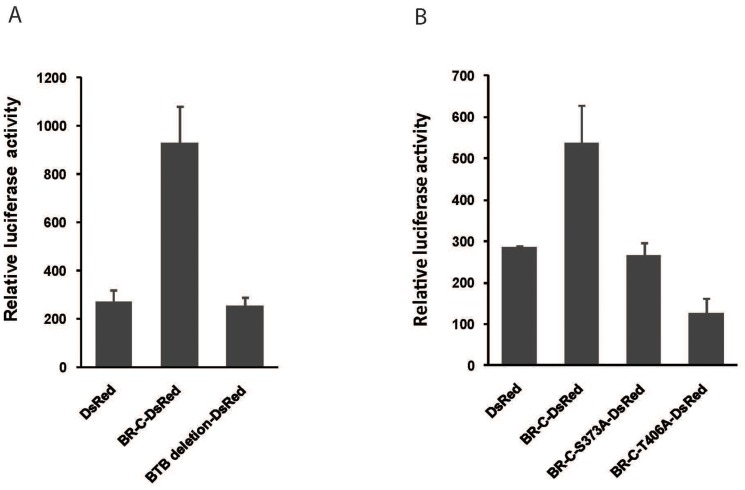
Both BTB domain deletion and PKC phosphorylation site mutants impaired the transcriptional activity of BR-C. (A) Effects of the deletion of the BR-C BTB domain on the transcriptional activity of BR-C. DsRed fused to BR-C lacking the BTB domain was co-transfected into BmN4 cells with a plasmid containing a luciferase reporter gene under the control of the promoter of *WCP10* gene, which is a direct target of BR-C. Luciferase activity was measured two days after transfection. Constructs containing either DsRed fused to full-length BR-C or DsRed alone were used as controls. (B) Effects of mutating the PKC phosphorylation sites in BR-C on the transcriptional activity of BR-C. The constructs containing BR-C with mutations at the phosphorylation sites S373 or T406 or non-mutated BR-C were each co-transfected into BmN4 cells with a plasmid containing a luciferase reporter gene under the control of the *WCP10* gene promoter. Luciferase activity was measured two days after transfection.

We reasoned that the disruption of BR-C nuclear localization might affect its transcriptional activity. To confirm this possibility, we selected the silkworm *WCP10* gene, which is transcriptionally regulated by the direct binding of BR-C to the *WCP10* promoter region [20]. We generated a construct that contains a luciferase reporter gene under the control of the *WCP10* promoter. This *WCP10* luciferase reporter vector was then co-transfected into BmN4 cells with expression plasmids containing DsRed fused to either full-length BR-C or partial BR-C lacking the BTB domain. Further analysis of luciferase activity showed that overexpressing full-length BR-C could elevate the activity of the *WCP10* promoter, compared with the DsRed alone control (Figure 8A). However, deleting the BTB domain completely abolished the transcriptional activity of BR-C toward the *WCP10* promoter (Figure 8A).

To further examine the importance of BR-C phosphorylation on its transcriptional activity, we analyzed the effects of the PKC phosphorylation site mutants of BR-C on the activity of the *WCP10* promoter. As shown in Figure 8B, compared to the full-length *BR-C* gene, both the Ser373 and Thr406 mutants of BR-C exhibited clearly decreased activities towards the *WCP10* promoter. These data, together with above-described findings, supported that the suppression of both BTB deletion and phosphorylation site mutations in BR-C on the transcriptional activation of target gene *WCP10* is likely due to their disruption on the nuclear import of BR-C.

## Discussion

The transcription factor BR-C is involved in controlling insect growth and development by mediating cross-talk between two endocrine hormones, ecdysone and juvenile hormone [3]. To date, the role of the DNA binding zinc finger domain of BR-C in response to endocrine hormones and transcriptional regulation of target gene expression has been well studied [20], [47], [48]. In addition to zinc fingers, insect BR-C proteins also features a BTB domain that is involved in protein-protein interactions [28]. However, the role of BTB domain in BR-C function remains unknown. In this study, we used yeast two-hybrid screening to identify RACK1 as an interacting partner for the silkworm BR-C protein by using the BTB domain as bait. Further examination revealed that this BTB domain-mediated interaction between BR-C and RACK1 plays an important role in the nuclear import and signaling of BR-C.

A large number of previous studies have shown that RACK1 primarily functions as a scaffold to recruit and interact with binding partners, and this interaction endows RACK1 with critical roles in regulating the transcription, translation, and cellular localization of RACK1-interacting proteins [45], [46], [49]. Consistent with these observations, our present data revealed a role for RACK1 in promoting the nuclear localization of BR-C in silkworm BmN4 cells. Furthermore, this role was mediated by the interaction of RACK1 with the BTB domain of BR-C. Because BR-C and RACK1 were localized to the nucleus and the cytoplasm, respectively, in silkworm BmN4 cells, we speculated that the interaction between BR-C and RACK1 most likely occurs in the cytoplasm. These data therefore strongly supported the notion that the interaction between BR-C and RACK1 is essential for the nuclear import of BR-C.

RACK1 has been shown to regulate posttranslational modification by recruiting activated PKC to its interacting partners, which in turn leads to the activation of these interacting partners via the PKC-mediated phosphorylation [37], [45]. For instance, it has been recently shown that the activity of the transcription factor ultraspiracle (USP) involved in insect ecdysone signaling is regulated by PKC-mediated phosphorylation [50], [51]. In addition, previous evidence has also demonstrated that the nuclear import of several proteins is modulated by their phosphorylation [52]–[55]. In fact, we predicted 12 PKC phosphorylation sites in BR-C and found that the mutations at only two sites, Ser373 and Thr406, could affect the nuclear import of BR-C. Taken together with the observation that the nuclear import of BR-C can be similarly disrupted by RNAi knockdown of the endogenous *PKC* gene in silkworm BmN4-SID1 cells, we concluded that the interaction between BR-C and RACK1 functions in a large part to recruit PKC to BR-C, such that BR-C is consequently phosphorylated by PKC and is then able to enter the nucleus.

Notably, the PKC phosphorylation sites in BR-C that are essential for the nuclear import of BR-C, namely, Ser373 and Thr406, are located in the two zinc finger domains, rather than in the BTB domain. Previous studies reported that the phosphorylation sites in several zinc finger-containing proteins are also located in the zinc finger domains [56], [57]. Therefore, our findings suggested that the interaction between the BTB domain of BR-C and RACK1 primarily contribute to recruit PKC and then help phosphorylate the phosphorylation sites in the zinc finger domains of BR-C.

**Figure 9:**
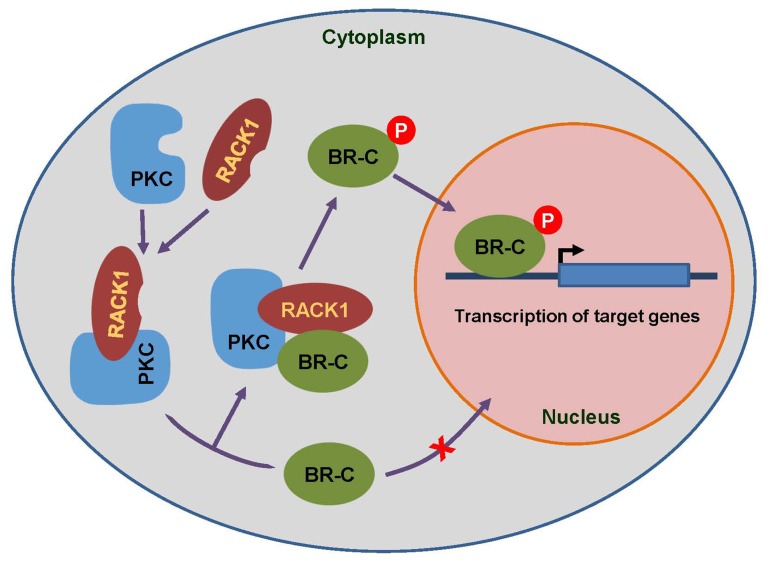
Proposed model for the nuclear import of BR-C upon interaction between BR-C and RACK1. The scaffolding protein RACK1 recruits and activates PKC in the cytoplasm. After being translated in the cytoplasm, BR-C interacts with RACK1 and is phosphorylated by RACK1-anchored PKC at amino acid residues Ser373 and Thr406. Phosphorylated BR-C then translocates into the nucleus and subsequently activates the transcription of its target genes.

Our examination also revealed that deleting the BTB domain and mutating the phosphorylation sites in BR-C impaired the transcriptional activation of target genes. Taken together with the other data presented here and the functions of RACK1 reported previously [45], we propose a potential model for the roles of the novel BR-C-interacting protein RACK1 in BR-C function. As summarized in Figure 9, RACK1 recruits active PKC to BR-C in the cytoplasm of the cells through its interaction with the BTB domain of BR-C. This interaction facilitates the phosphorylation of BR-C by PKC probably at Ser373 and Thr406. Phosphorylated BR-C then translocates to the nucleus to activate the transcription of BR-C target genes. To our knowledge, this study is the first report to describe a specific protein-protein interaction with insect BR-C and will be helpful for understanding the role of the BTB domain in the functions and signaling of BR-C in insects.

Unquestionably, the mechanism underlying BR-C phosphorylation remains to be deciphered in depth. For instance, it is enticing to determine whether Ser373 or Thr406 is the authentic phosphorylation sites in BR-C using liquid chromatography-tandem mass spectrometry (LC-MS/MS) analysis. In addition, given that BR-C is involved in the signaling of two key endocrine hormones, ecdysone and juvenile hormone [3], it is worthy to test whether and how BR-C phosphorylation is involved in modulating endocrine hormone signaling. Undoubtedly, future studies on BR-C phosphorylation are likely to provide new insights into the functions and signaling of BR-C in insects.

## Supporting Information

Figure S1 **Prediction of PKC phosphorylation sites in silkworm BR-C.** PKC phosphorylation sites in silkworm BR-C were predicted using the online NetPhosK program (http://www.cbs.dtu.dk/services/NetPhosK/). Based on a threshold score of 0.6, a total of 12 sites in the BR-C protein were predicted as PKC phosphorylation sites, including Ser (S) or Thr (T). All sites are highlighted in red. The BTB domain and the two zinc finger motifs are highlighted with blue and green boxes, respectively.
(TIF)

Figure S2 **Mutations at nine of the predicted PKC phosphorylation sites in silkworm BR-C have no effect on nuclear import of BR-C.** Among the predicted PKC phosphorylation sites in silkworm BR-C, in addition to Ser44 described in Figure 6, mutations to Ala (A) at nine sites also have no effect on nuclear localization of BR-C in BmN4 cells.
(TIF)

Table S1 **List of primers used in this study.**
(TIF)

Table S2 **List of plasmids, strains and cell lines used in this study.**
(TIF)
