# Supplementary figures and images for: Nuclear Import of Transcription Factor BR-C Is Mediated by Its Interaction with RACK1
Source: PLoS One. 2014 Oct 3;9(10):e109111. doi: 10.1371/journal.pone.0109111 (PMC4184850; doi:10.1371/journal.pone.0109111)

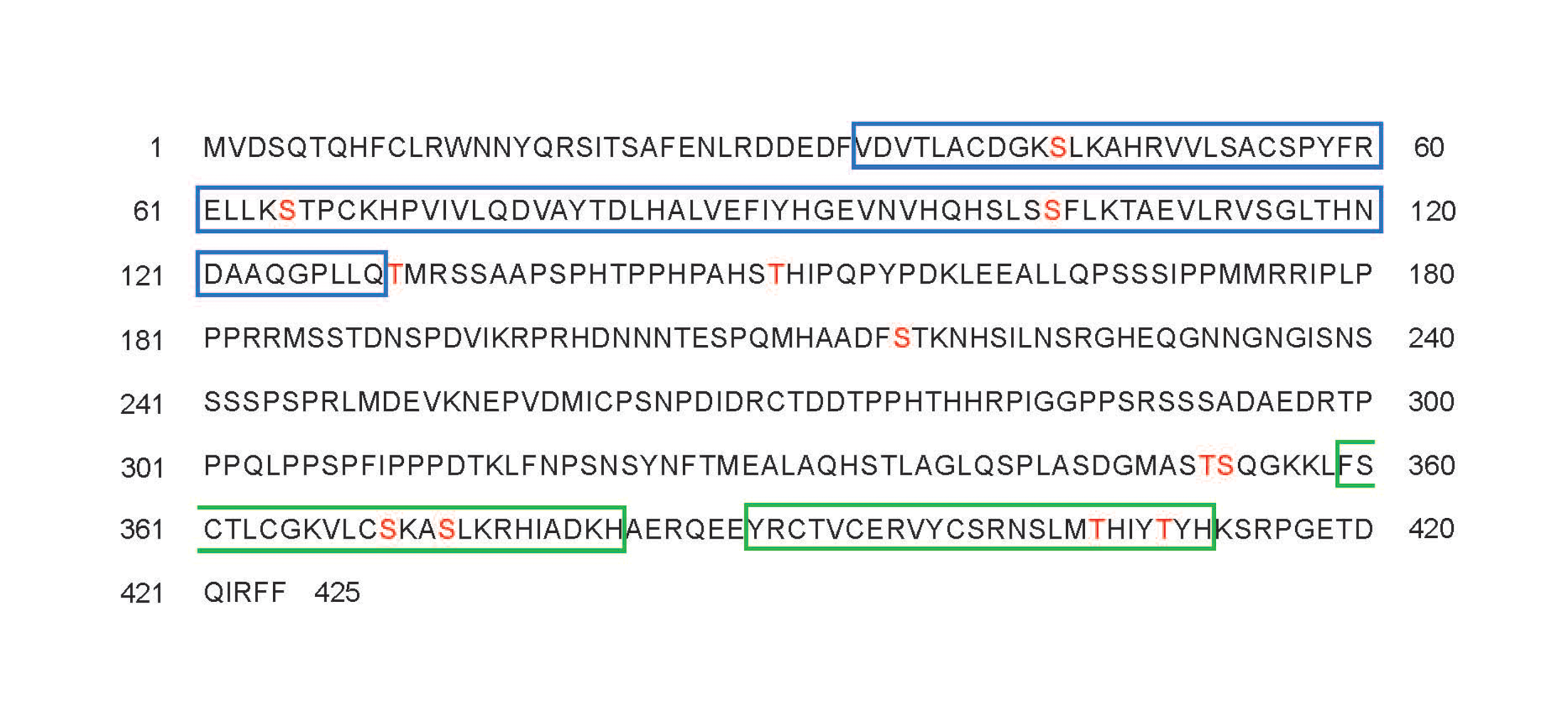

Supplement: Figure S1 — Prediction of PKC phosphorylation sites in silkworm BR-C. PKC phosphorylation sites in silkworm BR-C were predicted using the online NetPhosK program (http://www.cbs.dtu.dk/services/NetPhosK/). Based on a threshold score of 0.6, a total of 12 sites in the BR-C protein were predicted as PKC phosphorylation sites, including Ser (S) or Thr (T). All sites are highlighted in red. The BTB domain and the two zinc finger motifs are highlighted with blue and green boxes, respectively. (TIF) [file pone.0109111.s001.tif]

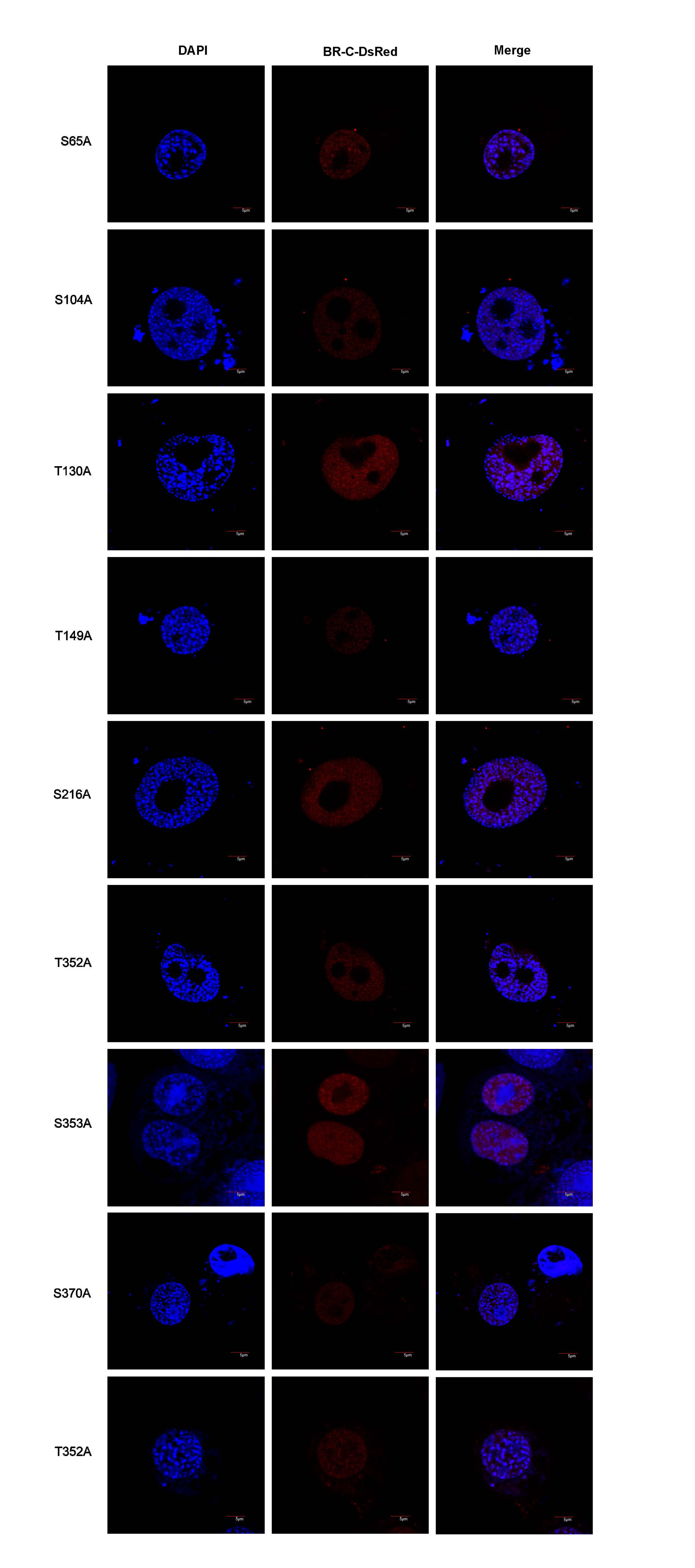

Supplement: Figure S2 — Mutations at nine of the predicted PKC phosphorylation sites in silkworm BR-C have no effect on nuclear import of BR-C. Among the predicted PKC phosphorylation sites in silkworm BR-C, in addition to Ser44 described in Figure 6, mutations to Ala (A) at nine sites also have no effect on nuclear localization of BR-C in BmN4 cells. (TIF) [file pone.0109111.s002.tif]

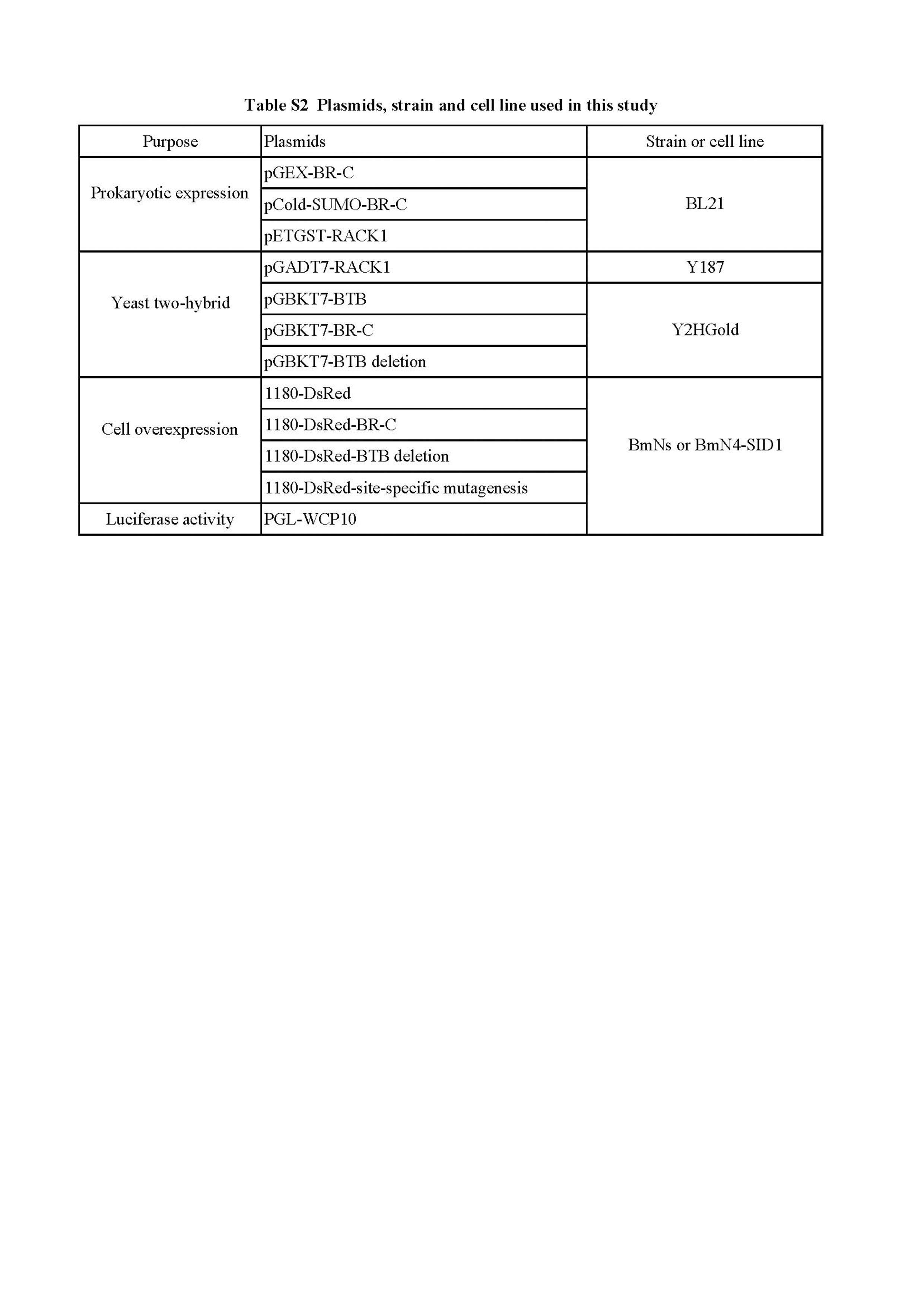

Supplement: Table S2 — List of plasmids, strains and cell lines used in this study. (TIF) [file pone.0109111.s004.tif]
